# Cerebellar hypoplasia caused by calcium/calmodulin-dependent serine protein kinase deficiency

**DOI:** 10.1016/j.mocell.2026.100347

**Published:** 2026-03-12

**Authors:** Katsuhiko Tabuchi, Emi Kouyama-Suzuki, Toru Yanagawa, Yoshinori Shirai

**Affiliations:** 1 Department of Molecular & Cellular Physiology, Shinshu University School of Medicine, 3-1-1 Asahi, Matsumoto, Nagano 390-8621, Japan; 2 Department of Oral and Maxillofacial Surgery, Faculty of Medicine, University of Tsukuba, Tsukuba 305-8575, Japan

**Keywords:** Calcium/calmodulin-dependent serine protein kinase, Calcium/calmodulin-dependent serine protein kinase-related disorders, Cerebellar hypoplasia, C-Jun N-terminal kinase inhibitor, Liprin

## Abstract

Calcium/calmodulin-dependent serine protein kinase (CASK) is an X-linked multidomain scaffolding protein originally identified as an intracellular binding partner for Neurexins, a family of presynaptic cell-adhesion molecules. Loss-of-function mutations in CASK cause microcephaly with pontine and cerebellar hypoplasia (MICPCH), a severe neurodevelopmental disorder predominantly affecting females. Although CASK has been implicated in synaptic organization and transcriptional regulation, the mechanisms underlying the cerebellar hypoplasia have remained unsolved. Recent studies using genetically engineered mouse models and cerebellar granule cell cultures suggest that CASK is essential for neuronal survival rather than for initial patterning in the cerebellum. These works further reveal that X-chromosome inactivation–driven mosaicism influences the pathology of this disorder and that CASK deficiency activates c-Jun N-terminal kinase (JNK) signaling. In this review, we integrate these findings with the synaptic cell-adhesion biology, in relevance to Neurexin–CASK interaction and role of CASK in cerebellar neuron survival and discuss emerging therapeutic implications for CASK-related disorders.

## Graphical Abstract

**Figure:**
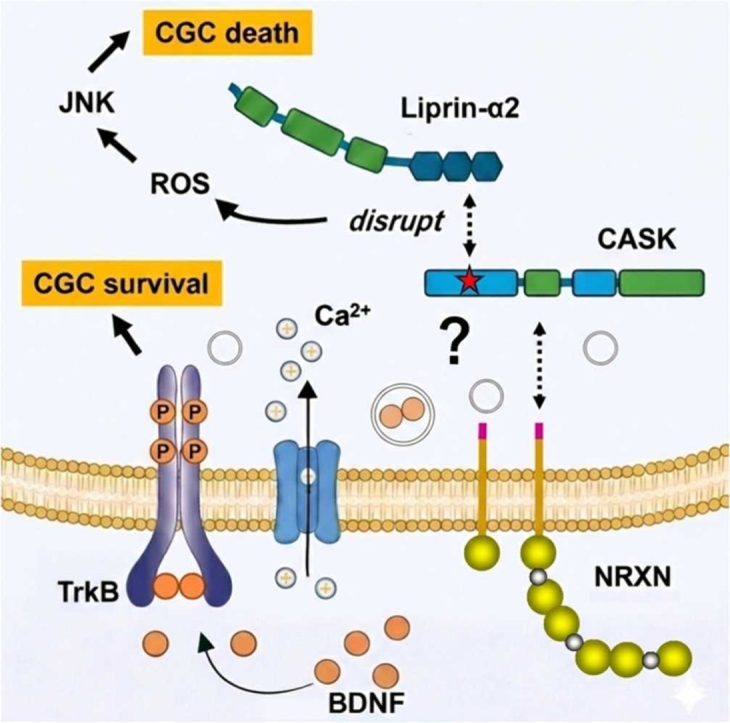

## INTRODUCTION: SYNAPTIC CELL-ADHESION MOLECULES AND CASK

In the central nervous system, synaptic cell-adhesion molecules are engaged in assembly, refinement, and long-term stability of neuronal circuits (Gerrow and El-Husseini, 2006, Missler et al., 2012, Washbourne et al., 2004). Through the binding specificity of these proteins, they ensure proper connectivity within complex neural networks (Giagtzoglou et al., 2009, Ko, 2012). Besides the adhesive function, synaptic cell-adhesion molecules also transmit intracellular signaling that regulates cytoskeletal dynamics, vesicle trafficking, local protein synthesis, and gene expression, thereby coupling synapse formation to neuronal differentiation, maintenance, and survival (Dalva et al., 2007, Duncan et al., 2021, Rudenko, 2017, Yang et al., 2014).

Among the diverse families of synaptic cell-adhesion molecules, neurexins may be the best characterized presynaptic organizers (Reissner et al., 2013, SÜdhof, 2017, Ushkaryov et al., 1992). Neurexins interact with certain families of postsynaptic ligands, such as neuroligins and leucine-rich repeat transmembrane proteins (Ichtchenko et al., 1995, Ko et al., 2009, SÜdhof, 2008, de Wit and Ghosh, 2016). Alternative promoters and extensive alternative splicing generate structural diversity of Neurexin proteins (Tabuchi and SÜdhof, 2002, Treutlein et al., 2014, Ullrich et al., 1995), enabling proper synapse formation through binding with appropriate postsynaptic ligands across neuronal subtypes and developmental stages. Besides the extracellular adhesion mechanism, neurexins recruit cytoplasmic scaffolding and signaling proteins through their short intracellular tail (Butz et al., 1998, Hata et al., 1996, Tabuchi et al., 2002).

Calcium/calmodulin-dependent serine protein kinase (CASK) is a membrane-associated guanylate kinase family protein that is originally identified as an intracellular binding partner for neurexins (Hata et al, 1996). CASK protein is comprised of a CaMK-like domain, 2 L27 motifs, a PDZ domain, an SH3 domain, and a guanylate kinase-like domain (Hata et al, 1996). This multidomain architecture enables CASK to function as a molecular hub that integrates signals from cell-surface adhesion molecules with cytoskeletal organization, synaptic vesicle exocytosis, and nuclear transcriptional programs (Butz et al., 1998, Hsueh et al., 2000, Hsueh, 2006, Mukherjee et al., 2008). CASK has been initially studied as a presynaptic protein as its interaction with neurexins, but subsequent works demonstrate its roles in the postsynapse or nucleus, where it cooperates with transcription factors such as TBR1 to regulate activity- and development-dependent gene expression (Hsueh et al., 2000, Hsueh, 2006). These findings highlight the pleiotropic characteristics of CASK and raise a question: which of these diverse functions are critical for the disease phenotype caused by CASK deficiency?

Synaptic contacts provide trophic and metabolic support, and disruption of adhesion-dependent signaling can compromise neuronal viability even in the absence of gross synaptic loss (Benson and Huntley, 2012, Uemura et al., 2010). The cerebellum, with its prolonged postnatal maturation and high dependence on activity-dependent survival cues, may be particularly vulnerable to perturbations in adhesion-mediated signaling (Guo et al., 2023, Uemura et al., 2022). This notion provides an important context for understanding why CASK deficiency preferentially affects the cerebellum (Südhof, 2018).

Microcephaly with pontine and cerebellar hypoplasia (MICPCH) represents the most severe phenotype associated with CASK deficiency (Najm et al, 2008). Clinically, MICPCH is characterized by congenital or early postnatal microcephaly, profound cerebellar and pontine hypoplasia, severe developmental delay, epilepsy, hypotonia, and progressive motor impairment (Hackett et al., 2010, Hayashi et al., 2017, Najm et al., 2008, Takanashi et al., 2012). The predominance of affected females indicates the importance of X-linked mosaicism as a result of X-chromosome inactivation in disease pathogenesis (Moog et al., 2015, Takanashi et al., 2012). Understanding how CASK deficiency leads to selective cerebellar vulnerability within this genetic context is therefore a major challenge for both basic neuroscience and translational research.

## CLINICAL AND GENETIC FEATURES OF CASK-RELATED CEREBELLAR HYPOPLASIA

Mutations in the CASK gene have been identified in patients who are diagnosed with a wide range of neurodevelopmental disorders. These mutations include nonsense and frameshift mutations that abolish normal protein production, splice-site mutations that disrupt the processing of transcripts, and missense variants that impair affinity to binding partner proteins (Mori et al, 2023). Accumulating clinical and genetic data have revealed a robust genotype-phenotype correlation (Hackett et al., 2010, Hayashi et al., 2017, Moog et al., 2015). Representative CASK mutations and their associated clinical phenotypes are summarized in Table 1. Mutations that result in a complete loss of CASK function are generally associated with cerebellar hypoplasia, whereas milder mutations, such as missense mutations, often retain cerebellar structure but can cause a partial set of complications of MICPCH syndrome, including X-linked intellectual disability, congenital nystagmus, epileptic encephalopathy, or late-onset ataxia (Hackett et al., 2010, Hayashi et al., 2017, Moog et al., 2015, Najm et al., 2008). These observations indicate that cerebellar hypoplasia is caused by the loss of function of CASK.

**Table 1:** Genotype-phenotype relationships in CASK-related disorders.

Major disease entity	Mutation type	Representative phenotype	Functional studies reported	Key references
Microcephaly with pontocerebellar hypoplasia (MICPCH)	Loss-of-function variants (nonsense, frameshift, deletions)	Severe intellectual disability, progressive microcephaly, pontocerebellar hypoplasia	Limited functional data; mainly clinical genetic studies	Najm et al (2008); Moog et al (2011); Moog et al (2015); Srivastava et al (2016)
MICPCH-like phenotypes associated with CaMK domain variants	Missense variants in the CaMK domain	Cerebellar hypoplasia and neurodevelopmental delay	Yes (structural and functional analyses of CASK-Liprin interaction)	Guo et al (2023); Tibbe et al (2022)
X-linked intellectual disability with nystagmus	Missense or splice variants	Mild to moderate intellectual disability±nystagmus	Yes (neuronal and mouse studies)	Hackett et al (2010); Moog et al (2015); Mori et al (2019)
Neurodevelopmental disorders linked to PSG supramodule variants	Missense variants in PDZ–SH3–GK supramodule	Variable neurodevelopmental phenotypes	Yes (biochemical studies showing reduced neurexin binding)	Tibbe et al (2025)
Complete CASK deficiency (mouse model)	Null allele	Neonatal lethality, cleft palate, synaptic dysfunction	Yes (cask knockout mouse studies)	Atasoy et al (2007)

Neuroimaging studies in patients with MICPCH have shown the vermis in the cerebellum, together with the pons and brainstem, is most prominently affected in many cases (Hayashi et al., 2017, Moog et al., 2011, Najm et al., 2008, Takanashi et al., 2012). Importantly, longitudinal imaging has shown that cerebellar volume can decrease over time, indicating that progressive neuronal loss contributes to the phenotype (Moog et al., 2011, Takanashi et al., 2012). These clinical observations suggest that cerebellar hypoplasia caused by CASK deficiency may be due to the impaired neuronal maintenance and survival during postnatal development.

## X-CHROMOSOME INACTIVATION AND CASK FUNCTION IN THE FOREBRAIN

Because complete loss of CASK is lethal in males (Atasoy et al, 2007), heterozygous female mice have been indispensable for dissecting CASK function *in vivo*. In females, X-chromosome inactivation generates a mosaic population of CASK-expressing and CASK-deficient neurons within the same brain region (Mori et al, 2019). This unique genetic situation provides a powerful tool to distinguish between cell-autonomous and non-cell-autonomous effects of CASK deficiency in the same brain.

Electrophysiological analyses in medial prefrontal cortex in acute cortical slices from heterozygous CASK knockout (CASK-hKO) mice reveal that CASK-deficient layer 5 pyramidal neurons exhibit a disrupted balance between excitatory and inhibitory synaptic inputs (Mori et al, 2019). This phenotype is attributable to reduced transcription of the NMDA receptor subunit GluN2B, whose gene expression is regulated by the nuclear CASK-TBR1 complex during development (Mori et al, 2019). Importantly, these synaptic abnormalities are restricted to CASK-deficient neurons and do not propagate to neighboring CASK-expressing cells, demonstrating a cell-autonomous effect (Mori et al, 2019). These studies establish that CASK plays a critical role in transcription-dependent synaptic maturation and highlight X-linked mosaicism as a determinant of circuit-level dysfunction.

Consistent with this forebrain phenotype in CASK-hKO mice, studies using human induced cortical excitatory neurons have shown that CASK loss does not compromise neuronal survival but instead leads to stage-dependent impairments in synaptic transmission and network synchronization, reinforcing the view that CASK deficiency in the forebrain primarily affects synaptic maturation rather than neuronal viability (McSweeney et al, 2022).

Although these forebrain phenotypes provide a compelling explanation for intellectual disability and epilepsy in CASK-related disorders, they are insufficient to explain the dramatic cerebellar hypoplasia characteristic of MICPCH syndrome. The investigation of CASK function in cerebellar neurons, particularly cerebellar granule cells, which represent the most abundant neuronal population in the brain, in CASK mutant mice has been conducted.

## CEREBELLAR HYPOPLASIA AS A CONSEQUENCE OF NEURONAL LOSS

CASK-hKO mice show cerebellar shrinkage (Guo et al., 2023, Mori et al., 2019, Srivastava et al., 2016). Histological studies demonstrate that the laminar formation of the cerebellum is largely preserved, whereas progressive thinning of the granule cell layer occurs in CASK-hKO and calb2-Cre-mediated CASK conditional knockout (CASK-cKO) mice in which CASK is removed in the post-migratory cerebellar neurons (Guo et al., 2023, Patel et al., 2022). In contrast, notable neuronal loss is not observed in the L7 promoter (Purkinje cell specific) or Math5 promoter (granule cell specific) dependent CASK-cKO mice (Srivastava et al, 2016).

In dissociated cerebellar granule cell cultures prepared from CASK-flox mice, acute deletion of CASK by infecting Cre recombinase-expressing lentivirus induces apoptotic cell death (Guo et al, 2023). The cell death is rescued by re-expression of wild-type CASK, indicating a requirement of CASK for the survival of postmitotic cerebellar neurons (Guo et al, 2023). Detailed structure-function analyses further clarify the molecular requirements for CASK-dependent neuronal survival. Patient-derived missense mutations within the CaMK domain of CASK—including R106P, L209P, R255C, and Y268H—fail to rescue cerebellar granule cell survival *in vitro* (Guo et al, 2023). These mutations selectively disrupt the interaction between CASK and another presynaptic scaffold protein Liprin-α2, while leaving interaction interfaces with other proteins intact, linking impaired CASK–Liprin-α2 coupling to cerebellar neuron loss (Guo et al, 2023).

Independent support for the CASK-Liprin-α2 interaction in pathogenesis is provided. Patient-derived CASK mutations affect Liprin-α2-dependent presynaptic organization in the transfected cell line model (Tibbe et al, 2022). Although this study does not directly represent cerebellar pathology, it establishes CASK-Liprin-α2 coupling as a key molecular interaction underlying presynaptic integrity, thereby reinforcing the pathogenic relevance of disrupted CASK-Liprin-α2 interactions identified in cerebellar granule cells.

At the structural level, studies have demonstrated that patient-derived variants affecting the PDZ–SH3–GK supramodule of CASK, which is cooperatively required for binding to the cytoplasmic tails of presynaptic adhesion partners such as Neurexins, highlight the significance of intact CASK scaffolding architecture for adhesion-dependent presynaptic organization (Tibbe et al, 2025). Together, these findings place CASK within a presynaptic cell-adhesion-associated complex that is essential for the maintenance of cerebellar neurons. This provides a possibility for adhesion-dependent survival mechanisms of cerebellar granule cells, as discussed below.

Recent work has shown that vertebrate-specific exons (19 and 20) within the CASK interdomain region undergo alternative splicing in both forebrain and hindbrain, and that splice-dependent inclusion alters the structural flexibility of the CASK C-terminus (Patel et al, 2024). These findings raise the possibility that regional differences in splice variant composition may modulate CASK-dependent signaling. However, because major splice isoforms are present in both cortex and cerebellum and quantitative differences appear modest (Patel et al, 2024), alternative splicing alone is unlikely to account for the marked vulnerability of cerebellar granule cells. Instead, splice-dependent structural modulation may fine-tune CASK interactions within region-specific circuit contexts.

## NEUREXINS AND CEREBELLAR GRANULE CELL SURVIVAL

**Fig. 1:**
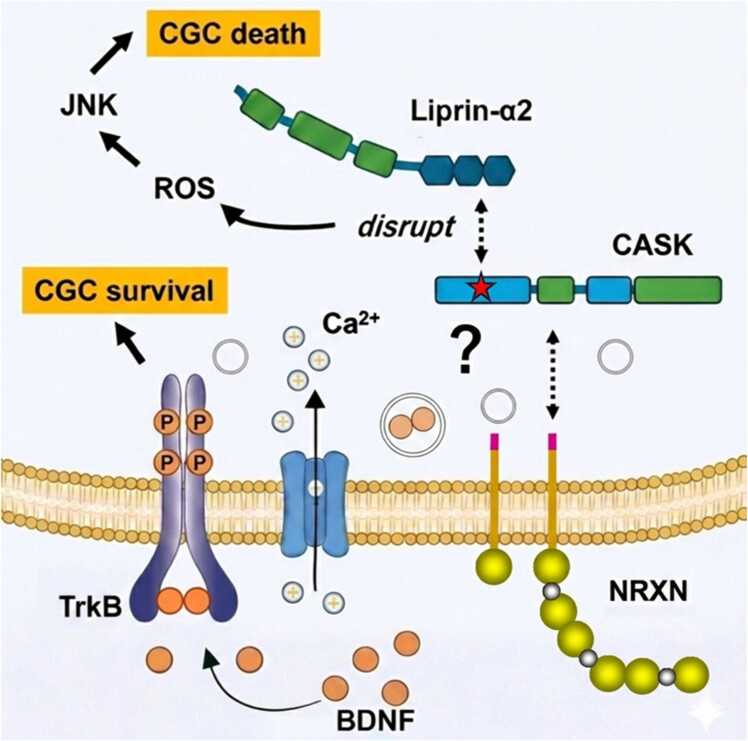
*Distinct presynaptic mechanisms regulating cerebellar granule cell survival.* Cerebellar granule cell (CGC) survival during postnatal development is supported by multiple presynaptic mechanisms. Neurexins (NRXNs) organize presynaptic-like structures in CGC axons and enable activity-dependent Ca²⁺ influx and autocrine secretion of brain-derived neurotrophic factor (BDNF), which activates TrkB signaling to promote CGC survival. In contrast, CASK interacts with the presynaptic scaffold protein Liprin-α2, and disruption of CASK-Liprin-α2 coupling activates stress-responsive pathways, including reactive oxygen species (ROS) accumulation and c-Jun N-terminal kinase (JNK) signaling, leading to CGC death. At present, it remains unclear whether these Neurexin-dependent and CASK-Liprin-α2-dependent mechanisms operate within a shared pathway or distinct processes that independently regulate CGC survival.

The link between synaptic cell-adhesion molecules and cerebellar hypoplasia is further strengthened by the study in the cerebellar granule cell-specific Neurexin triple knockout (Neurexin-TKO) mice. These mice exhibit severe cerebellar granule cell loss and cerebellar hypoplasia (Uemura et al, 2022). Neurexins have been implicated in calcium-dependent secretory granule exocytosis (Dudanova et al, 2006). Loss of neurexins impairs activity-dependent secretion of brain-derived neurotrophic factor (BDNF) from axons of cerebellar granule cells and this results in the cerebellar granule cell death in culture system (Uemura et al, 2022). Application of exogenous BDNF rescues neuronal survival, indicating that Neurexins support cerebellar granule cell viability by enabling intrinsic trophic signaling (Uemura et al, 2022). While parallel fiber-Purkinje cell synapses are formed and maintained through Neurexin-Cbln1-GluD2 transsynaptic interaction (Uemura et al, 2010), cerebellar granule cell survival may be independent of extracellular binding functions but mediated by the intracellular molecular interaction of Neurexins (Uemura et al, 2022). Given that CASK is a core intracellular binding partner for Neurexins, these findings raise the possibility that disruption of Neurexin- and CASK-associated presynaptic scaffolding may compromise neuronal survival programs in cerebellar neurons through shared molecular mechanisms. CASK forms an activity-dependent complex with neurexin and liprin-α, linking presynaptic adhesion molecules to active zone scaffolds (LaConte et al, 2016). Disruption of this axis may destabilize presynaptic organization and impair synaptic maintenance. In the cerebellum, where precise active zone architecture is essential for parallel fiber synapses, such destabilization may be particularly detrimental. We therefore propose that impaired Neurexin-CASK-Liprin-α coupling represents an upstream event that contributes to synaptic stress and subsequent degeneration of cerebellar granule cells. However, whether these pathways truly converge or operate through distinct processes remains questionable (Fig. 1).

## X-LINKED MOSAICISM AND SELECTIVE VULNERABILITY OF CEREBELLAR NEURONS

To address why cerebellar pathology is particularly severe in heterozygous females with CASK deficiency, mosaic patterns of X-chromosome inactivation are directly visualized using an X-linked GFP reporter strategy. For this purpose, the Hprt-eGFP mouse line, in which an EGFP expression cassette is knocked into the X-linked *hprt* locus (Kobayashi et al, 2016), was crossed with CASK-hKO mice to generate Hprt-eGFP; CASK-hKO trans-heterozygous female mice (Mori et al, 2025). In these mice, GFP-positive cells retain functional CASK that is inherited from the Hprt-eGFP allele, whereas the existence of the CASK-KO allele is exhibited as GFP-negative in CASK-deficient cells.

During early postnatal development, cerebellar granule cells exhibit an approximately equal mixture of GFP-positive and GFP-negative populations, consistent with random X-chromosome inactivation (Mori et al, 2025). Strikingly, this balanced mosaicism is progressively altered during postnatal maturation. Quantitative analyses reveal a marked reduction of GFP-negative (CASK-KO) granule cells over time, such that by adulthood the vast majority of surviving granule cells were CASK-positive (Mori et al, 2025). Purkinje cells display a different temporal profile. By postnatal day 6, CASK-deficient Purkinje cells are already largely absent, indicating that their elimination occurs extremely early during postnatal development. This early loss contrasts with the more gradual postnatal elimination observed in cerebellar granule cells and underscores cell-type–specific windows of vulnerability within the cerebellum (Mori et al, 2025). Importantly, this selective loss of CASK-deficient neurons is not accompanied by gross abnormalities in early cerebellar patterning, cell proliferation, or migration, indicating that CASK deficiency primarily influences neuronal survival rather than initial cerebellar development (Mori et al, 2025).

Previous studies using global and neuron-specific CASK-cKO mice have shown that CASK regulates postnatal cerebellar and cortical growth through mechanisms that are not strictly cell-autonomous, suggesting contributions from tissue-level or network-dependent processes (Patel et al., 2022, Srivastava et al., 2016). In this context, mosaic analyses provide critical cellular resolution, demonstrating that within a shared cerebellar environment, neurons lacking CASK are selectively lost.

Complementary experiments using hypomorphic or uniformly reduced CASK expression models showed substantially milder neuronal loss compared with mosaic animals (Mori et al, 2025), underscoring the importance of cellular context in determining neuronal vulnerability. The phenotypic differences between global or mosaic CASK deficiency and cell type-specific conditional knockout models likely reflect both temporal and cellular context (Srivastava et al., 2016, Patel et al., 2022). In female CASK heterozygous models, X-chromosome inactivation generates a mosaic cerebellar environment in which CASK-positive and CASK-deficient neurons coexist (Mori et al, 2019). Our recent analyses demonstrated that although approximately half of cerebellar granule cells initially lack CASK expression, CASK-deficient granule cells and Purkinje cells are selectively lost during postnatal development, resulting in a predominance of CASK-positive neurons in adulthood (Mori et al, 2025). This finding suggests a competitive survival mechanism in which relative cellular fitness influences neuronal maintenance (Mori et al, 2025). In contrast, L7- or Math5-Cre-mediated conditional deletion produces a more uniform cellular environment within targeted populations and does not fully recapitulate the mosaic competitive context (Srivastava et al, 2016). Moreover, the timing of Cre expression and the completeness of recombination may influence whether neurons experience CASK loss during a critical postnatal vulnerability window. Together, these observations support a model in which both cell-autonomous vulnerability and non-cell-autonomous competitive interactions contribute to cerebellar degeneration in CASK-related disorders. Consistent with this model, X-linked mosaicism creates a cerebellar environment in which CASK-deficient neurons are selectively eliminated during postnatal development, providing a mechanistic explanation for the progressive and female-biased cerebellar hypoplasia observed in MICPCH syndrome.

## STRESS SIGNALING AND JNK AS A THERAPEUTIC TARGET

Transcriptomic analyses of CASK-deficient cerebellar granule cells reveal robust activation of stress-responsive pathways, particularly those involving c-Jun N-terminal kinase (JNK) signaling and reactive oxygen species (Guo et al, 2025). Activation of JNK signaling is a well-established mediator of neuronal stress responses and apoptosis, suggesting that aberrant stress signaling contributes directly to cerebellar granule cell death following CASK loss (Figure 1).

Pharmacological inhibition of JNK using the selective inhibitor JNK-IN-8 markedly suppresses cerebellar granule cell death *in vitro* (Guo et al, 2025). Importantly, local administration of JNK-IN-8 into the cerebellum of mosaic CASK mutant mice reduces granule cell loss and ameliorates ataxic phenotypes *in vivo* (Guo et al, 2025). These findings position JNK signaling downstream of CASK deficiency and identify it as a tractable therapeutic target. Notably, JNK inhibitors are explored in other cerebellar degenerative conditions, raising the possibility that shared stress-response pathways underlie diverse forms of cerebellar vulnerability (Table 2) (Edamakanti et al., 2023, Harris et al., 2002, Li et al., 2021, Zanjani et al., 2013).

**Table 2:** Pharmacological and genetic evidence supporting JNK inhibition in models of cerebellar degeneration.

Study	Model	Inhibitor/manipulation	Main findings	Implications
Guo et al (2025)	*CASK*-deficient mouse (MICPCH model); cultured cerebellar granule cells	JNK-IN-8	CASK deficiency activated JNK signaling and increased ROS production. JNK-IN-8 suppressed ROS, prevented granule cell apoptosis, and improved cerebellar atrophy and motor performance *in vivo*.	First direct evidence that pharmacologic JNK inhibition rescues CASK-related cerebellar hypoplasia, supporting therapeutic use in MICPCH.
Edamakanti et al (2023)	*Spinocerebellar ataxia type 1 (SCA1)* mouse and human autopsy cerebellum	JNK pathway inhibitor (pharmacologic; blocked c-Jun phosphorylation)	Identified JNK-dependent c-Jun phosphorylation in Bergmann glia of SCA patients and SCA1 mice. JNK inhibition reduced glial inflammation and improved motor and histopathological phenotypes.	Established glial JNK activation as a pathogenic driver of SCA1; supports JNK inhibitors as potential therapy for spinocerebellar degeneration.
Zanjani et al (2013)	*Lurcher* (GluRδ2 mutation) cerebellar slice culture (Purkinje cell degeneration model)	SP600125 (JNK inhibitor)	JNK inhibition increased survival of both mutant and wild-type Purkinje cells in vitro.	Demonstrated that MAPK/JNK pathway overactivation contributes to Purkinje cell death, implicating JNK inhibition as neuroprotective.
Harris & Maroney (2002)	Cultured cerebellar granule neurons (low K⁺ apoptosis model)	CEP-1347 (KT7515) (MLK family inhibitor; upstream JNK blocker)	CEP-1347 completely blocked c-Jun phosphorylation and transcriptional induction, partially preventing apoptosis in vitro.	Provided molecular confirmation that JNK activation drives granule cell apoptosis; foundational mechanistic support for JNK inhibitors in cerebellar protection.
Li et al (2021)	*MAP3K DLK* and *LZK* overexpression mice (Purkinje cell-specific)	Genetic activation of JNK pathway	Overexpression of DLK caused rapid Purkinje cell apoptosis; LZK led to slower degeneration. Both activated JNK and caspase signaling independently.	Identified the DLK–LZK–JNK axis as the core degenerative cascade in Purkinje cells; supports targeting JNK signaling for therapeutic neuroprotection.

## CONCLUSIONS AND PERSPECTIVES

Cerebellar hypoplasia caused by CASK deficiency is best understood as a disorder of neuronal survival rooted in synaptic cell-adhesion signaling rather than a failure of early cerebellar patterning. Studies centered on the Neurexin-CASK-Liprin-α2 demonstrate that presynaptic adhesion complexes may play essential roles in sustaining cerebellar granule cell viability through activity-dependent trophic support. In females, X-chromosome inactivation generates a mosaic cerebellum in which CASK-deficient neurons are selectively eliminated *via* cell-autonomous mechanisms, providing a mechanistic explanation for progressive cerebellar hypoplasia in MICPCH syndrome.

Beyond CASK-related disorders, these findings have broader implications for the synaptic adhesion field. They suggest that synaptic cell-adhesion molecules are not only architects of synaptic connectivity but also regulators of neuronal fitness and survival. Disruption of adhesion-dependent intracellular signaling may therefore represent a unifying pathogenic principle linking synaptopathies and neurodegeneration. Future studies integrating synaptic biology with stress and survival signaling pathways, as well as emerging therapeutic approaches such as pathway-specific kinase inhibition or gene-based interventions, will be essential for translating these insights into disease-modifying strategies.

## Funding and support

This work was supported by grants KAKENHI 23H02575 (KT), the Grant-in-Aid for Transformative Research Areas (A) 23H04227 (KT), Grant-in-Aid for Transformative Research Areas (A) 21H05686 (KT), and SENSHIN Medical Research Foundation (KT).

## Author Contributions

KT wrote the paper. EKS, TY, and YS supervised the paper and provided feedback.

## Declaration of Competing Interests

The authors declare that they have no known competing financial interests or personal relationships that could have appeared to influence the work reported in this paper.
